# Prevalence and Correlates of Vitamin D Deficiency among Young South African Infants: A Birth Cohort Study

**DOI:** 10.3390/nu13051500

**Published:** 2021-04-29

**Authors:** Jabulani R. Ncayiyana, Leonardo Martinez, Elizabeth Goddard, Landon Myer, Heather J. Zar

**Affiliations:** 1 Division of Epidemiology & Biostatistics, School of Public Health and Family Medicine, Faculty of Health Sciences, University of Cape Town, Cape Town 7925, South Africa; landon.myer@uct.ac.za; 2 Division of Public Health Medicine, School of Nursing and Public Health, College of Health Sciences, University of KwaZulu-Natal, Durban 4000, South Africa; 3 Department of Epidemiology, School of Public Health, Boston University, Boston, MA 02118, USA; leomarti@bu.edu; 4 Department of Paediatrics and Child Health, Red Cross War Memorial Children’s Hospital, and SA-MRC Unit on Child & Adolescent Health, University of Cape Town, Cape Town 7700, South Africa; liz.goddard@uct.ac.za (E.G.); heather.zar@uct.ac.za (H.J.Z.)

**Keywords:** vitamin D deficiency, infancy, breastfeeding, maternal HIV, South Africa

## Abstract

Early-life vitamin D deficiency is associated with adverse child health outcomes, but the prevalence of vitamin D deficiency and its correlates in infants remains underexplored, particularly in sub-Saharan Africa. We aimed to investigate the prevalence of vitamin D deficiency and its correlates among young infants in South Africa. This study included 744 infants, aged 6–10 weeks from the Drakenstein Child Health Study, a population-based birth cohort. Infants were categorized into distinct categories based on serum 25(OH)D concentration level including deficient (<50 nmol/L), insufficient (50–74 nmol/L), and sufficient (≥75 nmol/L). Using multivariable Tobit and logistic regression models, we examined the correlates of serum 25(OH)D_3_ levels. The overall prevalence of vitamin D deficiency was 81% (95% confidence intervals (CI]) 78–83). Multivariable regression analysis showed that serum 25(OH)D_3_ concentration was independently associated with study site, socioeconomic status, and sex. Birth in winter and breastfeeding were the strongest predictors of lower serum 25(OH)D_3_ concentration levels. Compared to non-breastfed children, children breastfed were at higher risk of vitamin D deficiency (AOR, 1.96; 95% CI, 1.04–3.67) and breastfeeding for more than one month was associated with greater likelihood of vitamin D deficiency (AOR, 5.40; 95% CI, 2.37–12.32) and lower vitamin D concentrations (−16.22 nmol/L; 95% CI, −21.06, −11.39). Vitamin D deficiency in infants is ubiquitous, under-recognised, and strongly associated with season of birth and breastfeeding in this setting. Nutritional interventions with vitamin D supplementation in national health programs in low- and middle-income countries are urgently needed to improve early-life vitamin D status in infants.

## 1. Introduction

Vitamin D deficiency is a re-emerging public health problem in children across many low- and middle-income countries [1]. Vulnerability to vitamin D deficiency in early-life is commonly attributed to increased micronutrient requirements for child growth, particularly bone development, inadequate nutrient intake, or limited exposure to sunlight [2]. In addition to increased risk of growth impairment and rickets in children, vitamin D deficiency has also been associated with numerous other adverse child health outcomes such as respiratory tract infections [3,4,5], asthma, allergies [6,7], and type 1 diabetes mellitus [8]. There is also growing evidence to suggest that the impact of vitamin D deficiency in the first few years of life may extend to adulthood, when vitamin D deficiency is associated with cancer, cardiovascular disease, or infections including COVID-19 [9,10,11]. Vitamin D and its derivatives are also used in the treatment of various medical conditions [12]. Understanding the epidemiology of vitamin D deficiency in children, especially young infants, is key to the prevention of a broad range of diseases across the lifespan.

Several studies have reported a high prevalence of vitamin D deficiency among infants globally including in the Middle East [13,14,15], Europe [16,17], Asia [18] and sub-Saharan Africa [19,20,21]. Reported prevalence of vitamin D deficiency among infants in sub-Saharan Africa varies from 30% to 76% [19,20,21]. However, there is only one published study of vitamin D status in South African infants, reporting a prevalence of vitamin D deficiency of 33% [22]. Infant risk factors for low vitamin D levels in sub-Saharan Africa included maternal vitamin D status, maternal education, season of birth, low birthweight, urban residence or breastfeeding [19,20,21,22]. 

Despite many benefits of breastfeeding, breastmilk has been shown to have low vitamin D levels [23]. Thus, breastfed infants are at higher risk of vitamin D deficiency as they may not get adequate vitamin D intake from the breastmilk [23]. As a result, oral supplementation of 400 IU per day of vitamin D is recommended for all breastfeeding infants in developed countries [24,25,26]. In Australia and New Zealand targeted testing for vitamin D levels in population groups who are at risk of vitamin D deficiency, including breastfed infants, is also recommended [26]. In South Africa, there is high frequency of breastfeeding initiation and suboptimal complementary feeding practices [27,28]. Data from our Drakenstein Child Health Study show that about 86% of newborns are breastfed and 60% continued breastfeeding at the age of nine months [27]. There is an urgent need for evidence to identify high risk paediatric populations in low- and middle-income countries, who may benefit from targeted vitamin D deficiency and supplementation.

Given the reported high prevalence of vitamin D deficiency among infants and its adverse health outcomes, estimating the prevalence of vitamin D deficiency and understanding contributing factors during early infancy is critical in designing effective interventions. In this study, we investigated the prevalence of vitamin D deficiency in infants in the first two months of life and explored correlates with vitamin D concentrations and deficiency in a population-based birth cohort in South Africa. Furthermore, we examined the association between breastfeeding and 25(OH)D_3_ levels. We hypothesize that vitamin D deficiency will be high in this population and that breastfed young infants would have lower 25(OH)D_3_ levels compared to young infants who were not breastfed.

## 2. Materials and Methods

### 2.1. Study Setting and Participants

The Drakenstein Child Health Study is a prospective birth cohort in a peri-urban area outside Cape Town, South Africa. The Drakenstein area has a latitude of 33° S, an elevation of 120 m and an average temperature of 6.8 °C in winter. A detailed description of the study design has been published previously [29]. Briefly, mothers in the second trimester of pregnancy were enrolled from two public health clinics (TC Newman and Mbekweni) serving distinct communities over three consecutive years. All births occurred at a central public hospital and were attended by a member of the study team; mother-child pairs were followed from birth through childhood. An initial study visit occurred at 6–10 weeks coinciding with a well-baby visit for immunisation. This population is characterised by high rates of poverty and unemployment. Most people live in informal or crowded housing conditions, and levels of alcohol consumption and smoking are high [30]. However. there is a strong primary health care program including antenatal care, HIV, prevention of mother-to-child transmission and expanded program for immunisation [30]. An infant serum sample was taken at six–ten weeks of age and subsequently biobanked. Inclusion in this study was based on the availability of a stored serum sample at this timepoint.

### 2.2. Vitamin D Status

Vitamin D status was assessed by serum 25-hydroxyvitamin D (25(OH)D) concentration (nmol/L). Three serum 25(OH)D metabolites; (nonepimeric: 25(OH)D_3_, epimeric: 3-epi-25(OH)D_3_, and 25(OH)D_2_) were measured at Vitas AS (Oslo, Norway; a reference laboratory in Europe with a Vitamin D External Quality Assessment Scheme certificate) from specimens stored at −80 °C using liquid chromatography-tandem mass spectrometry [31]. Previous evidence has shown the stability of 25(OH)D and its metabolites in stored serum samples for more than 10 years [32]. The assay’s lower limits of quantification (LLQs) for nonepimeric 25(OH)D_3_, 3-epi-25(OH)D_3_, and 25(OH)D_2_ were 5, 3, and 5 nmol/L, respectively.

### 2.3. Covariates

Explanatory variables included infant and maternal factors. Infant variables include age, sex, anthropometry measures at age 6–10 weeks: height-for-age z score (HAZ) and weight-for-age z score (WAZ) at 6 weeks of age, maternal HIV exposure, prematurity (defined as gestational age < 37 weeks), feeding practices (exclusive breast feeding, exclusive formula or mixed), season of birth, and study sites. Season of birth was categorized into summer (December–February), autumn (March–May), winter (June–August), and spring (September–November). Maternal variables included maternal age, self-reported antenatal smoking and educational level, parity and household socio-economic status assessed using a validated composite score comprising 4 variables comprising asset ownership, household income, employment and education [30].

### 2.4. Statistical Analysis

The study population characteristics were summarised using frequencies and proportions for categorical or binary variables, means and standard deviations for symmetric continuous distributions, and medians and interquartile ranges for asymmetric distributions. Descriptive statistics were tabulated for the two study communities and compared across locations using chi-square tests for proportions and *t*-tests for means, as appropriate. Serum 25(OH)D concentrations were summarised using means, standard deviations, medians and interquartile ranges. We also reported serum 25(OH)D concentrations by season of birth, stratified by study site. Although there is no consensus on 25(OH)D levels for vitamin D deficiency [33], the following categories were used to define vitamin D status based on the literature: vitamin D deficiency (<50 nmol/L), insufficiency (50–74 nmol/L) and sufficiency (≥75 nmol/L), to compare results with other studies [33]. The proportions of infants with vitamin D deficiency, insufficiency, and sufficiency were calculated to describe the prevalence of vitamin D deficiency.

The association between assessed predictors and vitamin D status was assessed using Tobit regression models. Tobit regression models were employed because 25(OH)D_3_ as a continuous outcome was left censored at the assay’s LLQs of 5 nmol/L and was therefore skewed [34]. Only variables with a *p*-value below 0.2 were included in multivariable Tobit regression analysis. We also explored possible study site effects on the correlates of 25(OH)D levels by adding an interaction term between site and the following characteristics: smoking status, socioeconomic status, sex, HIV-exposure, and infant feeding practices. Only significant interaction terms were included in full model. Separate Tobit regression models were fitted for Mbekweni and TC Newman sites.

Finally, we assessed the relationship between breastfeeding and low vitamin D concentration, using Tobit regression models as described above. We also evaluated the relationship between breastfeeding and vitamin D deficiency using multivariable logistic regression models. We grouped participants into those with vitamin D deficiency and all other participants into a referent group. We repeated these analyses assessing whether infants breastfeed for >1 month and <1 month were more likely to have vitamin D deficiency or low vitamin D concentrations (compared to infants not breastfed). A linear test for trend was completed for both models using orthogonal polynomial contrasts. All statistical tests were 2-sided at alpha = 0.05. All analyses were conducted in Stata version 16 (StataCorp Inc., College Station, TX, USA).

## 3. Results

**Figure 1:**
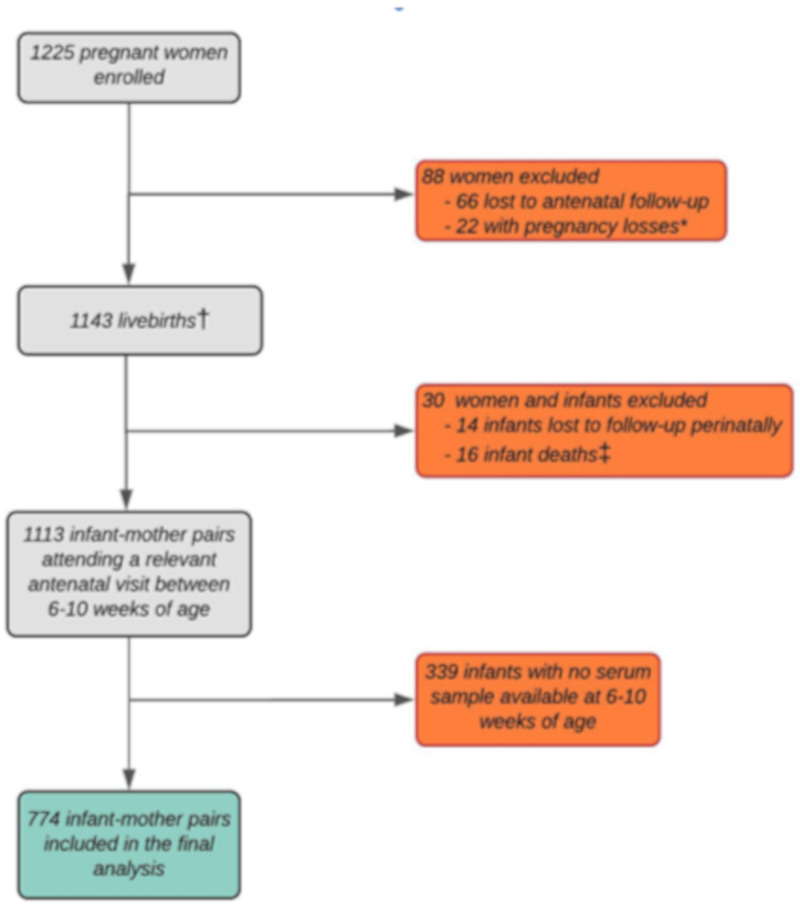
Study flow diagram of eligibility and enrollment of mothers and infants in the Drakenstein Child Health study, Cape Town, South Africa. * Loss of pregnancy due to miscarriage, stillbirth, or intrauterine death (23 infants (including one set of twins)). † Including four pairs of twins and one set of triplets. ‡ No postnatal data collected.

Between 5 March 2012, and 31 March 2015, 1225 pregnant women were recruited and enrolled in the birth cohort. 88 women were excluded due to being lost to antenatal follow-up (*n* = 66) and pregnancy losses (*n* = 22). This resulted in 1143 live births (four sets of twins and one triplet), with 1113 infants followed up at 6–10 weeks. Amongst these, 339 did not have a serum sample available; therefore, 774 mother-infant pairs were available for this analysis (Figure 1). Characteristics of 369 infants excluded did not differ significantly from those included (Table S1).

Among the included population, 166 (22%) mothers were HIV-infected while only one (<1%) infant was HIV-infected, due to a highly effective antenatal antiretroviral therapy programme. The median maternal age was 26 (IQR, 22–31) years and about one in four mothers were smokers by self-report (*n* = 206; 27%). Breastfeeding was initiated in the majority of the cohort at birth (*n* = 718; 93%) (Table 1). The mean weight-for-age z-score at six weeks of age was −0.39 (standard deviation, −1.15, 0.33). The median age at the time of testing for vitamin D status was 54 days (IQR, 48–58).

The study sites were similar in terms of infant age at testing, season of birth, sex, maternal education, and the proportion born preterm (Table 1). In comparison with Mbekweni infants, TC Newman infants had lower weight-for-age z-scores, were more likely to have been breastfed and exposed to tobacco smoke, while in Mbekweni, a higher proportion of infants were HIV-exposed (38%).

Overall, vitamin D deficiency was 80.6% (95% CI, 77.7–83.3); 1.8% (95% CI, 1.0–3.0) of infants were vitamin D sufficient while 17.6% (95% CI, 15.0–20.4) were insufficient. Infants from TC Newman had higher prevalence of vitamin D deficiency compared to infants from Mbekweni (87% versus 75%, *p* < 0.0001). Median serum 25(OH)D_3_ and 3-epi-25(OH)D_3_ concentrations were 37.4 nmol/L (IQR, 25.8–47.3) and 4.5 nmol/L (IQR, 1.5–6.1), respectively. 24 (3.1%) infants had undetectable levels of serum 25(OH)D_3_ while 318 (41.1%) had undetectable levels of serum 3-epi-25(OH)D_3_ (Table 1). The majority of infants had undetectable levels of serum 25(OH)D_2_. Only one infant (0.1%) had serum 25(OH)D_2_ concentrations above lower limit of quantification for 25(OH)D_2_ of 5 nmol/L. Vitamin D deficiency also differed by sex (84% versus 76%, *p* = 0.010), season of birth (*p* = 0.013), and breastfeeding practices (82% versus 66%, *p* =0.004).

**Figure 2:**
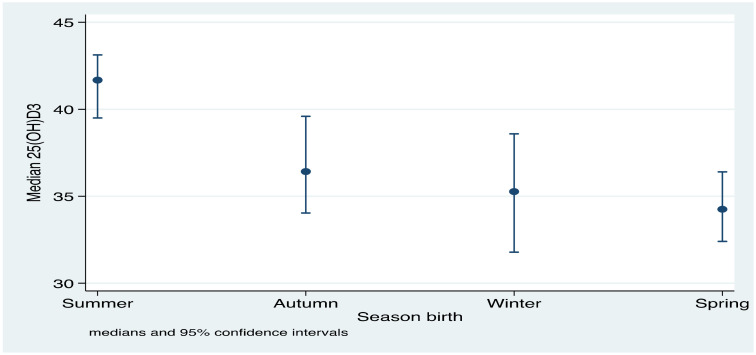
Median serum 25(OH)D_3_ concentration by season of birth. Season of birth was categorized into summer (December–February), autumn (March–May), winter (June–August), and spring (September–November).

Statistically significant seasonal differences in 25(OH)D_3_ concentrations were observed (Figure 2). Infants born in summer had median 25(OH)D_3_ concentrations of 41.7 nmol/L (IQR, 33.1–49.7), higher than infants born in winter (median, 35.3 nmol/L; IQR, 14.9–45.0) and spring months (median, 34.3 nmol/L; IQR, 25.5–43.9). There was a statistical trend based on season of birth starting in summer, autumn, winter, and spring (P_trend_ < 0.0001; Figure 2).

**Figure 3:**
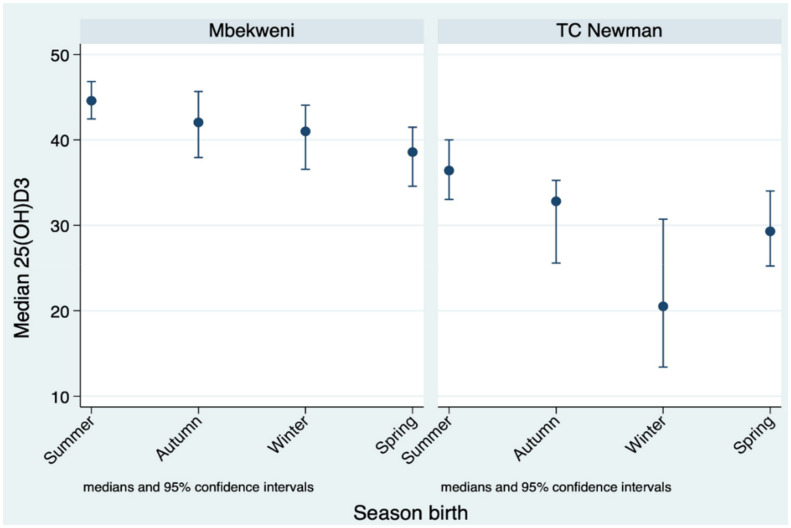
Median serum 25(OH)D_3_ concentration by season of birth and study site. Season of birth was categorized into summer (December–February), autumn (March–May), winter (June–August), and spring (September–November). Study site (Mbekweni and TC Newman).

When examined by site, infants from TC Newman had lower median 25(OH)D_3_ concentrations compared to infants from Mbekweni (32.1 vs 41.8 nmol/L, *p* < 0.0001). Median 25(OH)D_3_ concentrations in infants born in winter were 41.0 nmol/L (95% CI, 44.0–36.5) and 20.5 nmol/L (95% CI, 30.7–13.4) in Mbekweni and TC Newman, respectively. Infants from TC Newman also have more pronounced 25(OH)D_3_ concentrations variation with an average difference of 5.3 nmol/L between the seasons compared with 2.0 nmol/L for infants from Mbekweni (Figure 3).

In univariable analysis among both study sites, serum 25(OH)D_3_ concentration was associated with site, season of birth, smoking status, socioeconomic status, sex, and breastfeeding, which were negatively associated with serum 25(OH)D_3,_ while age, WAZ and HAZ at six weeks of age, and HIV exposure were positively associated with serum 25(OH)D_3_ concentration levels. For every 1 unit increase in WAZ and HAZ at six weeks of age, 25(OH)D_3_ increased by 1.46 nmol/L and 1.07 nmol/L, respectively.

In univariable analyses, there were clear differences in risk factors for vitamin D deficiency among the study sites. When stratifying by site, serum 25(OH)D_3_ concentration was significantly associated with season of birth, socioeconomic status, age, gender, and breastfeeding in Mbekweni. However, in TC Newman, serum 25(OH)D_3_ concentration was significantly associated with season of birth, socioeconomic status, maternal HIV, and infant feeding practices.

Multivariable Tobit regression models, overall and stratified by study site, associated with vitamin D concentrations are shown in Table 2. After adjustment for confounders in a multivariable analysis, several risk factors were identified. Season of birth remained statistically associated with vitamin D concentrations (Table 2). Compared to children born during the summer, children had lower vitamin D concentration if they were born during autumn (−5.54 nmol/L, 95% CI, −8.61 to −2.48), winter (−9.29 nmol/L, 95% CI, −12.37 to −6.21), and spring (−6.70 nmol/L, 95% CI, −9.88 to −3.53) months. In addition, the study site TC Newman was also associated with lower vitamin D concentrations (−8.57; 95% CI, −11.41 to −5.75) compared with Mbekweni.

In a multivariable analysis adjusted for sex of child, study site, maternal HIV, socioeconomic status, season of birth, and weight-for-age z-scores, breastfeeding was consistently associated with both vitamin D deficiency and lower vitamin D concentrations (Table 3).

Breastfed infants were almost at two times higher risk of vitamin D deficiency (AOR, 1.96; 95% CI, 1.04–3.67). There was a dose-response relationship between vitamin D deficiency and breastfeeding duration; compared to children not breastfed, there was a higher likelihood of vitamin D deficiency among infants breastfed <1 month (AOR, 2.26; 95% CI, 1.03–4.93) and infants breastfed ≥1 month (AOR, 5.40; 95% CI, 2.37–12.32). A multivariable Tobit regression adjusted for these variables found similar associations. Breastfed infants had vitamin D concentrations −9.74 nmol/L (95% CI, −14.72 to −4.76) lower than infants that were not breastfed. Again, there was a consistent gradient of vitamin D concentrations and breastfeeding duration; compared to children not breastfed, there were lower vitamin D concentrations among infants breastfed <1 month (adjusted coefficients, −4.91 nmol/L; 95% CI, −9.68 to −0.15) which was even greater among infants breastfed ≥1 month (adjusted coefficients, −16.22 nmol/L; 95% CI, −21.06 to −11.39). There was a statistically significant trend by duration of breastfeeding for both vitamin D deficiency and vitamin D concentrations (both P_trend_ < 0.0001).

## 4. Discussion

This study in a population-based cohort has shown an extraordinarily high prevalence (81%) of vitamin D deficiency in infants in this part of South Africa. Overall, vitamin D status was very poor indicated by many infants with undetectable levels of 3-epi-25(OH)D_3_ and 25(OH)D_2_ metabolites and only 2% being vitamin D sufficient. Notably, boys had lower 25(OH)D levels than girls while breastfed infants were more likely to be both vitamin D deficient and have lower vitamin D concentrations. 25(OH)D_3_ concentration was independently associated with site, season of birth, socioeconomic status, age, and sex. Infants in this cohort were generally healthy, so detection of vitamin D deficiency so early in life was clinically unrecognised, suggesting that routine vitamin D screening and supplementation should be considered in low- and middle-income countries such as South Africa.

Previous studies investigating the prevalence of vitamin D deficiency among infants have shown heterogeneous results. A smaller study of 291 infants in South Africa [22] found a lower prevalence (33%) of vitamin D deficiency in neonates on cord blood measures, with a higher mean serum 25(OH)D_3_ concentration of 41 nmol/L compared to 36 nmol/L in our study [22]. However, the high prevalence of vitamin D deficiency in our study is similar to recent reports from elsewhere in Africa. One study of 581 infants with serum 25(OH)D levels assessed at six weeks in Tanzania reported a 76% prevalence of vitamin D deficiency [20]. This study used a cut-off of <50 nmol/L, similar to that used in our study [20]. Another cross-sectional Tanzanian study of 446 infants aged two weeks found a 60% prevalence of vitamin D deficiency using a cut-off of <50 nmol/L [19]. Differences in timing of measurements, cut-off values to define vitamin D deficiency, methods used for vitamin D measurement, and study populations may explain some of the variability in these studies. For example, the South African study of a lower prevalence of vitamin D deficiency used a more conservative cut-off of <30 nmol/L to define vitamin D deficiency [22].

Infant WAZ at six weeks of age was positively associated with 25(OH)D concentration and this remained after adjustment in a multivariable model. Previous studies have shown variable results with a few consistent with ours [35,36], while a study among Tanzanian infants did not find an association between vitamin D status and WAZ or HAZ at six weeks of age [20]. One study conducted in Ecuadorian children found that underweight or stunted children were more likely to have lower serum 25(OH)D concentration [35]. WAZ is a nutrition indicator and therefore these results indicate concurrent vitamin D deficiency with malnutrition. We found that WAZ was positively associated with vitamin D concentrations, but this was of borderline statistical significance in our study.

Our finding of low 25(OH)D concentration among infants born in winter or spring compared to those born in summer is consistent with two studies conducted in Middle Eastern and Asian infants [18,37] and two in sub Saharan African [20,22]. In South Africa, infants born in winter had four times the odds of vitamin D deficiency [22]. Another study in South African children aged 10 years found that vitamin D status was associated with season [38]. Low 25(OH)D concentration in winter may be explained by limited skin exposure to sunlight in that season.

Breastfeeding was the strongest predictor of low 25(OH)D concentrations and vitamin D deficiency. There was a dose-response association, with children breastfed for more than one month having more than five times the likelihood of vitamin D deficiency compared to children not breastfed. Children breastfed for <1 month were still more likely to be vitamin D deficient, but at lower levels. Our findings are consistent with several other studies from high-income settings [39,40]. In Tanzania, infants exclusively breastfed at six weeks of age were twice as likely to be vitamin D deficient [20]. This may reflect inadequate amounts of vitamin D in breastmilk if mothers who are breastfed are undernourished. Alternatively, secondary factors such as maternal HIV may have influenced this association. However, when adjusting for these variables the association between breastfeeding and vitamin D concentration and deficiency remained, as well as a dose-response by duration of breastfeeding. In TC Newman, almost all infants (99.7%) were breastfed, and more than half (54.3%) were breastfed for more than one month. This may explain the vitamin D level heterogeneity between the two study sites.

This analysis is strengthened by the detailed data on maternal and infant health, including comprehensive anthropometric data in a large cohort. Further, the laboratory that measured the 25(OH)D levels was an accredited European reference laboratory. Our use of the 50-nmol/L cut-off to define vitamin D deficiency is conservative and provides the most accurate description of the burden of vitamin D deficiency in South African infants to date. Previous investigators used lower cut-offs, which may have underestimated the magnitude of the problem [22]. A limitation of this study is that maternal vitamin D status and vitamin supplementation during pregnancy, which may influence infant vitamin D status, were not assessed. However, most mothers were young (median, 25 years of age) and generally healthy; most who were HIV-infected were well controlled on antiretroviral therapy (ART).

## 5. Conclusions

In summary, our results suggest that vitamin D deficiency in infants is highly prevalent in South Africa and more common than previously recognised. We observed strong associations of 25(OH)D concentrations at six–ten weeks with breastfeeding and season of birth, and children who breastfed for longer durations were more likely to be vitamin D deficient, suggesting that micronutrient supplementation among these children may be indicated. This high prevalence of vitamin D deficiency in the early weeks of life is a cause for concern given the potential impact on child and adult health. Routine screening for vitamin D deficiency and supplementation should be considered in infants in low- and middle-income countries. In South Africa, vitamin D supplementation is currently not routinely provided; our data suggest that this should be instituted early in primary health care, especially in exclusively breastfed infants. Further research on the health trajectory of children in relation to vitamin D deficiency is needed, and nutritional interventions to improve infants’ vitamin D status are urgently needed.

## Figures and Tables

**Table 1:** Characteristics of mothers and infants in the Drakenstein Child Health Study by study site.

Variables	Mbekweni (*n* = 411)	TC Newman (*n* = 363)	*p*-Value	Total (*n* = 774)
Maternal characteristics				
Median Age, years (IQR)	27 (23–32)	25 (21–29)	<0.001	26 (22–31)
Age group, years				
<20	36 (8.8)	53 (14.6)		89 (11.5)
20–24	122 (29.7)	131 (36.1)	<0.001	253 (32.7)
25–29	108 (26.3)	101 (27.8)		209 (27.0)
≥30	145 (35.3)	78 (21.5)		223 (28.8)
Parity				
0	137 (33.3)	152 (41.9)		289 (37.3)
1	162 (39.4)	122 (33.6)	0.048	284 (36.7)
≥2	112 (27.3)	89 (24.5)		201 (26.0)
Education				
Primary	32 (7.8)	26 (7.2)		58 (7.5)
Some Secondary	227 (55.2)	186 (51.2)	0.296	413 (53.4)
Completed Secondary	124 (30.2)	132 (36.4)		256 (33.1)
Some Tertiary	28 (6.8)	19 (5.2)		47 (6.1)
HIV positive	157 (38.2)	9 (2.5)	<0.001	166 (21.4)
Smoking in pregnancy	20 (4.9)	162 (44.6)	<0.001	182 (23.5)
Season birth				
Summer (Dec–Feb)	123 (29.9)	97 (26.7)		220 (28.4)
Autumn (Mar–May)	100 (24.3)	95 (26.2)	0.539	195 (25.2)
Winter (June–Aug)	104 (25.3)	85 (32.4)		189 (24.4)
Spring (Sept–Nov)	84 (20.4)	86 (23.7)		170 (22.0)
Socioeconomic Status				
Lowest	124 (30.2)	60 (16.5)		184 (23.8)
Moderate Low	108 (26.3)	87 (24.0)	<0.001	195 (25.2)
Moderate high	98 (23.8)	100 (27.5)		198 (25.6)
High	81 (19.7)	116 (34.0)		197 (25.4)
Infant characteristics				
Weight-for-age z score	−0.16 (−0.92 to 0.51)	−0.63 (−1.30 to 0.06)	<0.001	−0.39 (−1.15 to 0.33)
Height-for-age z score	−0.72 (−1.63 to −0.16)	−0.97 (−1.89 to −0.17)	0.002	−0.83 (−1.75 to −0.01)
Female	206 (50.1)	160 (44.1)	0.093	366 (47.3)
Prematurity (<37 weeks)	55 (13.4)	43 (11.8)	0.521	98 (12.7)
Breastfeeding initiated	356 (86.6)	362 (99.7)	<0.001	718 (92.8)
Vitamin D status				
25(OH)D_3_				
Mean 25(OH)D_3_ (SD)	41.2 (15.3)	31.0 (17.2)	<0.001	36.4 (17.0)
Median 25(OH)D_3_ (IQR)	41.8 (32.8–49.9)	32.1 (18.2–42.1)	<0.001	37.4 (25.8–47.3)
Sufficient 25(OH)D_3_ (≥75 nmol/L)	9 (2.2)	5 (1.4)	<0.001	14 (1.8)
Insufficient 25(OH)D_3_ (50–74 nmol/L)	92 (22.4)	44 (12.1)		136 (17.6)
Deficient 25(OH)D_3_ (<50 nmol/L)	310 (76.4)	314 (86.5)		624 (80.6)
3-epi-25(OH)D_3_				
Mean 3-epi-25(OH)D_3_ (SD)	4.6 (3.5)	4.4 (3.9)	0.550	4.5 (3.7)
Median 3-epi-25(OH)D_3_ (IQR)	3.9 (1.5–6.2)	3.5 (1.5–5.8)	0.550	3.7 (1.5–6.1)

Abbreviations: IQR, interquartile range.

**Table 2:** Multivariable Tobit regression analyses of factors associated with serum 25(OH)D_3_ levels.

Variables	Mbekweni		TC Newman		Overall	
	Coefficient	95% CI	Coefficient	95% CI	Coefficient	95% CI
Maternal characteristics						
Smoker	0.14	−6.64 to 6.92	−1.32	−4.93 to 2.30	−1.05	−4.09 to 1.99
Season birth						
Summer (December–February)	1	Ref	1	Ref	1	Ref
Autumn (Mar–May)	−4.77	−8.71 to −0.83	−6.52	−11.33 to −1.71	−5.54	−8.61 to −2.48
Winter (June–August)	−6.43	−10.32 to −2.53	−12.51	−17.44 to −7.58	−9.29	−12.37 to −6.21
Spring (September–November)	−7.53	−11.65 to −3.41	−5.94	−10.89 to −1.00	−6.70	−9.88 to −3.53
Socioeconomic status						
Lowest	1	Ref	1	Ref	1	Ref
Moderate Low	−2.80	−6.72 to 1.12	−0.13	−5.71 to 5.45	−1.87	−5.09 to 1.35
Moderate high	−3.89	−7.73 to −0.06	0.87	−4.72 to 6.47	−1.89	−5.08 to 1.31
High	−2.08	−6.23 to 2.07	−2.09	−7.55 to 3.37	−2.80	−6.05 to 0.45
Infant characteristics						
Male sex	−3.77	−6.68 to −0.87	−1.92	−5.48 to 1.65	−3.01	−5.27 to −0.75
Study Site						
Mbekweni	…	…	…	…	1	Ref
TC Newman	…	…	…	…	−8.57	−11.41 to −5.74
WAZ at 6 weeks of age *	1.28	−0.04 to 2.61	0.89	−0.86 to 2.64	1.09	0.03 to 2.16
HIV exposure	−1.88	−5.27 to 1.52	−12.49	−24.28 to −0.71	−2.82	−6.23 to 0.59
Breastfeeding initiated	−9.17	−14.02 to −4.31	−13.66	−48.91 to 21.59	−9.75	−14.79 to −4.71

* WAZ: weight-for-age z score.

**Table 3:** Vitamin D deficiency and concentrations among infants breastfed and not breastfed in the Drakenstein Child Health Study.

	Adjusted Odds Ratio(95% CI), *p*-Value †		
**Vitamin D Deficiency**			
Not breastfed	1	Referent	…
Breastfed	1.96	1.04–3.67	0.036
Not breastfed ‡	1	Referent	…
Breastfed < 1 month	2.26	1.03–4.93	0.041
Breastfed ≥ 1 months	5.40	2.37–12.32	<0.0001
	**Adjusted Coefficient (95% CI), *p*-Value †**		
**Vitamin D concentration, nmol/L**			
Not breastfed	1	Referent	…
Breastfed	−9.74	−14.72, −4.76	<0.0001
Not breastfed ‡	1	Referent	…
Breastfed < 1 month	−4.91	−9.68, −0.15	0.043
Breastfed ≥ 1 months	−16.22	−21.06, −11.39	<0.0001

† All models are adjusted for sex of the child, study site, maternal HIV, socioeconomic status, season of birth, and weight-for-age z-scores. Odds ratios and 95% confidence intervals were calculated using multivariable logistic regression models. Regression coefficients and 95% confidence intervals were calculated using multivariable Tobit regression models. ‡ A linear test for trend using orthogonal polynomial contrasts was calculated with a *p*-value < 0.0001 across all four values.

## Data Availability

Data cannot be shared publicly because of ethical conditions with which study investigators are obliged to comply. Access to the project data is restricted to nominated investigators approved by the University of Cape Town Human Research Ethics Committee, as per the consent document. Interested, qualified researchers may request to access this data by contacting the Drakenstein Child Health Study (via lesley.workman@uct.ac.za) to submit a formal data use request and ensure required ethical approval received prior to use.

## Supplementary Materials

The following are available online at https://www.mdpi.com/article/10.3390/nu13051500/s1, Table S1: Comparison of 774 infants included versus 369 infants excluded in the Vitamin D study, Cape Town, South Africa.
