# Supplementary material for: Prevalence and Correlates of Vitamin D Deficiency among Young South African Infants: A Birth Cohort Study
Source: Nutrients. 2021 Apr 29;13(5):1500. doi: 10.3390/nu13051500 (PMC8146842; doi:10.3390/nu13051500)
Supplement: Supplementary file 1 [file nutrients-13-01500-s001.zip › nutrients-1140959-supplementary.pdf]

Supplementary Materials

Table S1. Comparison of 774 infants included versus 369 infants excluded in the Vitamin D study, Cape Town, South Africa.

**Variables**	**Included in vitamin D study (*n*=774)**	**Excluded in the vitamin D study (*n*=369)**
Study site		
Mbekweni	411 (53.1)	223 (60.4)
TC Newmann	363 (46.9)	146 (39.6)
Female	366 (47.3)	188 (51.0)
HIV Exposed	166 (21.4)	82 (22.4)
Prmaturity (< 37 weeks)	98 (12.7)	96 (26.0)
Season birth		
Summer (Dec–Feb)	220 (28.4)	68 (18.2)
Autumn (March–May)	195 (25.2)	86 (23.3)
Winter (June–August)	189 (24.4)	117 (31.7)
Spring (Sept– Nov)	170 (22.0)	99 (26.8)
Mother's education		
Primary	58 (7.5)	28 (7.6)
Some Secondary	413 (53.4)	196 (53.1)
Completed Secondary	256 (33.1)	119 (32.2)
Some Tertiary	47 (6.1)	26 (7.1)
Breastfeeding intiated	718 (92.8)	329 (90.4)
Socioeconomic status		
Lowest	184 (23.8)	90 (24.4)
Moderate Low	195 (25.2)	101 (27.4)
Moderate high	198 (25.6)	92 (24.9)
High	197 (25.4)	86 (23.3)
